# Dynamic Color Transform Networks for Wheat Head Detection

**DOI:** 10.34133/2022/9818452

**Published:** 2022-02-01

**Authors:** Chengxin Liu, Kewei Wang, Hao Lu, Zhiguo Cao

**Affiliations:** Key Laboratory of Image Processing and Intelligent Control, Ministry of Education, School of Artificial Intelligence and Automation, Huazhong University of Science and Technology, Wuhan, China

## Abstract

Wheat head detection can measure wheat traits such as head density and head characteristics. Standard wheat breeding largely relies on manual observation to detect wheat heads, yielding a tedious and inefficient procedure. The emergence of affordable camera platforms provides opportunities for deploying computer vision (CV) algorithms in wheat head detection, enabling automated measurements of wheat traits. Accurate wheat head detection, however, is challenging due to the variability of observation circumstances and the uncertainty of wheat head appearances. In this work, we propose a simple but effective idea—dynamic color transform (DCT)—for accurate wheat head detection. This idea is based on an observation that modifying the color channel of an input image can significantly alleviate false negatives and therefore improve detection results. DCT follows a linear color transform and can be easily implemented as a dynamic network. A key property of DCT is that the transform parameters are data-dependent such that illumination variations can be corrected adaptively. The DCT network can be incorporated into any existing object detectors. Experimental results on the Global Wheat Detection Dataset (GWHD) 2021 show that DCT can achieve notable improvements with negligible overhead parameters. In addition, DCT plays an important role in our solution participating in the Global Wheat Challenge (GWC) 2021, where our solution ranks the first on the initial public leaderboard, with an Average Domain Accuracy (ADA) of 0.821, and obtains the runner-up reward on the final private testing set, with an ADA of 0.695.

## 1. Introduction

Wheat is one of the principal cereal crops, playing an essential role in the human diet [1]. However, the growth of the world population and global climate change significantly threaten the supply of wheat [2]. To ensure sustainable wheat crop production, breeders need to identify productive wheat varieties by constantly monitoring many wheat traits. Among traits of interest, wheat head density, i.e., the number of wheat heads per unit area, is a key adaptation trait in the breeding process. It is closely related to yield estimation [3], stress-tolerant plant variety discovery [4], and disease resistance [5]. A natural way to estimate wheat head density is to detect every wheat head in a sampled area. In practice, wheat head density estimation still largely relies on human observation in the traditional breeding process, which is inefficient, tedious, and error-prone [6]. To meet the need of efficient measurement of wheat traits, it is required to develop machine-based techniques for automated wheat head detection.

**Figure 1:**
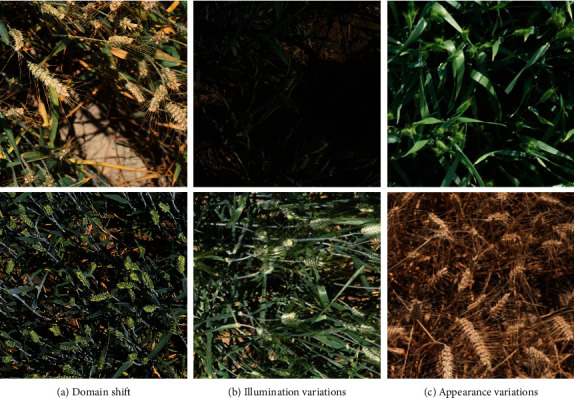
Some examples show the challenges of in-filed wheat head detection: (a) domain shift due to different locations; (b) illumination variations due to different observation conditions; (c) wheat head appearance variations due to different growth stages.

With the prevalence of affordable camera platforms (e.g., unmanned aerial vehicles and smartphones), in-filed image-based wheat head detection emerges as a potential solution to replace tedious manual observation. It enables automated measurements of wheat traits and therefore relieves the burden of human efforts. To develop efficient and robust detection algorithms, a large and diverse wheat head dataset is necessary. However, most existing wheat head datasets [3, 4, 6] are far from satisfactory. The limited number of images and genotypes cannot guarantee the robustness of CNN models in a new environment. In addition, inconsistent labeling protocols between different datasets impede the comparison of detection methods. To tackle the issues above, the Global Wheat Head Detection dataset [5, 7] is proposed. Based on this dataset, two sessions of the Global Wheat Challenge (GWC) have been held in the Computer Vision Problems in plant phenotyping workshops (CVPPP2020 [8] and CVPPA 2021 [9]), aimed at encouraging practitioners to develop robust algorithms. The hosting of GWC 2020 and GWC 2021 has attracted a large cohort of practitioners with computer vision backgrounds. With active contributions from competitors around the world, GWC has made an important step toward a robust solution to wheat head detection. Nevertheless, the nature of in-filed images renders wheat head detection a challenging task. As shown in Figure 1, there exist several visual challenges:

- *Domain shift*. Wheat head images acquired at different locations are diverse, leading to severe domain shifts. For example, the GWHD dataset covers genotypes from various countries, such as Europe, Australia, and Asia.
- *Illumination variations*. Since in-filed images are captured with ground-based platforms and cameras, illumination varies significantly under different observation conditions, especially under blazing sunlight.
- *Appearance variations*. Wheat heads exhibit distinct appearances at different developmental stages, e.g., wheat heads are green at the postflowering stage but turn yellow at the ripening stage.
- *Degraded images*. Natural conditions like wind may result in occluded images, making it hard to distinguish wheat heads.

Notice that some challenges above not only appear in wheat head detection but also occur in generic object detection. Fortunately, due to the emergence of large-scale datasets [10, 11] and high-performance graphics processing units (GPUs), deep learning has significantly advanced the progress of generic object detection [12–15]. Therefore, some challenges can be well addressed. For example, the powerful representation capability of convolutional neural networks (CNNs) [16–18] can mitigate the impact of appearance variations. By deploying heavy data augmentation during training, CNNs can adapt to degraded images to some extent. Despite the remarkable progress that has been achieved in generic object detection, some unique challenges in wheat head detection remain unsolved, e.g., domain shifts and illumination variations.

Recently, much effort has been made to wheat head detection [4, 6, 19]. Hasan et al. [4] apply Region-based Convolutional Neural Networks (R-CNN) for wheat spike detection, achieving high detection accuracy. Madec et al. [6] investigate two deep learning methods for wheat ear density estimation, i.e., FasterRCNN [13] and TasselNet [20], finding that FasterRCNN is more robust when the wheat ear is at the high maturity stage. Although previous studies report competitive results, the intrinsic challenges in wheat head detection are still overlooked, which impedes the progress of developing robust algorithms.

To address the aforementioned challenges, we propose the idea of dynamic color transform, aiming to adapt the CNN model to different illumination and domains. This idea is motivated by the observation that an appropriate treatment of color cues can greatly benefit wheat head detection, particularly in alleviating false negatives. Specifically, we present an analysis of the impact of the color channel and propose to deal with colors with dynamic color transform (DCT). The DCT is in the same spirit of recent dynamic networks [21, 22] that enable date-dependent inference. For example, the DCT follows a linear color model that dynamically generates 2 parameters to modulate the color of the input image.

We evaluate our method on the GWHD 2021 dataset. In particular, we validate the effectiveness of two formulations of DCT, i.e., a regression-based DCT and a classification-based DCT, and show that DCT is not sensitive to the hyperparameters chosen. Moreover, we initiate DCT on four different backbone networks, including MobileNetV2 [23], ShuffleNetV2 [23], ResNet18 [16], and ResNet34 [16]. Notably, the ResNet18- [16] based DCT network can operate 1024 × 1024 images at around 142 fps. Experimental results demonstrate that the use of DCT can help to achieve state-of-the-art performance of wheat head detection, with the validation ADA of 0.802 and the testing ADA of 0.657. DCT plays an important role in our competition entry in the GWC 2021, where we finally obtain the runner-up reward.

Our main contributions include the following:

- We investigate the impact of the color channel and observe that modifying the color channel of the input image can improve detection results
- We introduce a DCT network based on our observation and show that DCT can obtain notable improvements with negligible parameters overhead
- Our method reports state-of-the-art results on the GWHD 2021 dataset and achieves the runner-up performance on the Global Wheat Challenge 2021

The preliminary conference version of this work [24] appeared in the International Conference on Computer Vision (ICCV) Workshop—CVPPA 2021 (https://cvppa2021.github.io/). In this paper, we make the following extensions. First, we further investigate a classification-based formulation to model color transform. Second, we systematically explore the design of the DCT network, providing practical references to the agriculture and plant science community. Third, we further conduct substantial experiments and analyses to demonstrate the effectiveness of our method and to justify the rational of our design choice.

## 2. Related Work

### 2.1. Object Detection in Computer Vision

Object detection, a fundamental task in computer vision, has witnessed remarkable progress in recent years. In the era of deep learning, object detection is typically divided into two paradigms: two-stage detection and one-stage detection. The former formulates detection as a coarse-to-fine process, while the latter predicts the object in one step. FasterRCNN [13] is a classical two-stage object detector, which unifies object proposal, feature extraction, and bounding box regression. Specifically, a Region Proposal Network (RPN) is introduced to enable nearly cost-free region proposals. Then, a box refinement module is followed after RPN, outputting final predictions. To improve the efficiency of FasterRCNN, much effort has been made like cascade detection [25], position-sensitive regression [26], and feature pyramid [17]. In contrast to two-stage detection that consists of proposal generation and verification, one-stage detection outputs objects directly. You Only Look Once (YOLO) [27] is the first deep learning-based one-stage detector. It divides an image into separate regions and predicts the objects in each region simultaneously, therefore achieving fast inference speed. Despite being efficient, it suffers from localization errors and low recall. To address these issues, YOLOv2 [28] introduces several ideas to obtain better performance, such as batch normalization [29], high-resolution classifier, anchor boxes, fine-grained features, and multiscale training. A new architecture DarkNet therefore is proposed, which achieves promising results and maintains fast inference. Subsequently, YOLOv3 [30] presents some updates on YOLOv2. Several changes in the network design decorate the detection model, such as multiscale predictions and a stronger backbone. Further, Bochkovskiy et al. [12] empirically investigate the combinations of different features that are said to improve CNN accuracy. Based on the investigation, a new edition—YOLOv4—is presented. It integrates a bunch of new features (e.g., Cross-Stage-Partial (CSP) connections [18], path aggregated network (PAN) [31], and mosaic data augmentation), achieving state-of-the-art results. Built upon YOLOv4, Scaled-YOLOv4 proposes a network scaling method that modifies the depth, width, resolution, and structure of the detection network, aimed at maintaining the best trade-off between speed and accuracy.

Benefiting from the recent progress of object detection, DCT builds upon Scaled-YOLOv4. It is worth mentioning that our DCT is generic and is capable of cooperating with other object detectors.

### 2.2. Wheat Head Detection in Plant Phenotyping

In recent years, computer vision-based approaches have attracted great attention in crop detection [6, 19, 32, 33]. In particular, several methods [3, 4, 6, 19, 34] have been developed for wheat head detection. As wheat heads exhibit unique texture, i.e., the spatial arrangement of color or intensity in a specific region, Qiongyan et al. [3] proposes to leverage law texture energies for wheat spike detection. By incorporating texture features into a neural network, it achieves high classification accuracy. Following this idea, Narisetti et al. [34] adopts wavelet amplitude as the input image and suppresses nonspike structures using a Frangi filter. The improved method obtains more reliable results on European wheat plants. Another line of research focuses on leveraging the power of CNNs. Hasan et al. [4] presents a specifically designed deep learning model, i.e., Region-based Convolutional Neural Networks (R-CNN), for wheat spike detection. With the high-quality spike dataset, the R-CNN model achieves favorable detection accuracy. Madec et al. [6] investigate two deep learning methods, i.e., FasterRCNN [13] and TasselNet [20], in wheat ear density estimation. The results show that FasterRCNN is more robust than TasselNet when the wheat ear is at a high maturity stage. To reduce the labeling cost in cereal crop detection, Chandra et al. [19] proposes a point supervision-based active learning approach, saving more than 50% of the labeling time. In addition, synthesizing datasets [35] is also an appealing way to tackle the lack of large-scale training data.

In contrast to previous studies, we aim to develop high-performance detectors for wheat head detection by addressing illumination variations.

### 2.3. Dynamic Networks

Recently, dynamic networks emerge as a new research topic in deep learning. In contrast to conventional deep neural networks [16, 36] where the computational graphs and parameters are fixed, dynamic networks enable data-dependent inference where parameters or network architecture can be adapted conditioned on the input. A typical line of research in dynamic networks is to adapt network parameters to the input and to produce dynamic features. In the context of image classification, Spatial Transformer Networks (STNs) [37] allow the spatial manipulation of features via a differentiable data-dependent module, which makes neural networks robust to translation, scale, and rotation. Sharing a similar spirit, deformable convolutional networks [38, 39] perform irregular spatial sampling with learnable offsets and therefore achieve promising results on object detection and semantic segmentation. Apart from spatial transform, another solution is to reweight features with soft attention. The commonly used attention mechanisms include channel-wise attention [40], spatial-wise attention [41], or both [42]. Akin to soft attention, IndexNet [21, 22] is proposed to deal with the downsampling/upsampling stage in deep networks.

Our DCT is related to dynamic networks in the sense that it predicts color transform parameters based on the input. Different from previous studies, DCT manipulates the input image rather than the feature and therefore can easily cooperate with existing object detectors.

## 3. Materials and Methods

### 3.1. Global Wheat Head Detection Dataset

**Figure 2:**
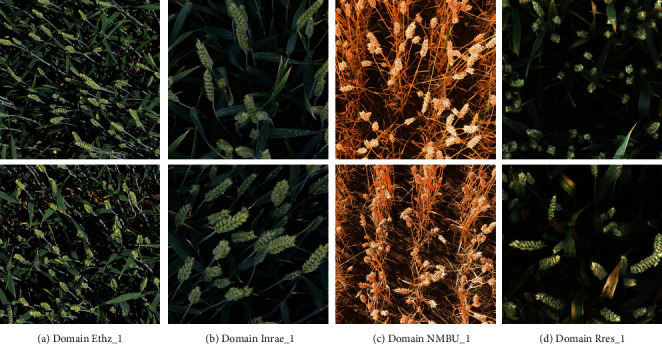
Example wheat head images from different domains: (a) domain Ethz_1 acquired in Switzerland; (b) domain Inrae_1 acquired in France; (c) domain NMBU_1 acquired in Norway; (d) domain Rres_1 acquired in the UK.

In this work, we adopt a recent Global Wheat Head Detection dataset 2021 [5, 7] as experimental data. The RGB images in the GWHD 2021 dataset are collected between 2015 and 2020 by 16 institutions distributed across 12 countries, covering genotypes from Europe, Africa, Asia, Australia, and North America. Since the GWHD dataset contains wheat heads across several developmental stages, e.g., postflowering and ripening stages, a definition of “subdataset” is introduced to help researchers to investigate the impact of each developmental stage. Specifically, a “subdataset” defines a domain, which is formulated as a consistent set of images captured under the same experimental and acquisition conditions.  Figure 2 shows examples of images from different domains. Notice that the images are acquired with various ground-based phenotyping platforms and cameras at the nadir-viewing direction, resulting in diverse image properties. For example, the platforms used by different institutions include spidercam, gantry, tractor, cart, and handheld.

To assemble the images from different “subdatasets,” a manual inspection is first conducted to eliminate the invalid images that contained unclearly visible wheat heads. Next, the original images are split into 1024 × 1024 squared patches. Each patch contains around 20 to 60 wheat heads, and a few heads will cross the patch edges. Following the standard object detection annotation paradigm, each wheat head is labeled by drawing a bounding box on a web-based labeling platform. The GWHD 2021 dataset hence is composed of these annotated squared patches, containing 3657 training images, 1476 validation images, and 1373 test images. It is worth mentioning that GWHD 2021 is used by the Global Wheat Challenge 2021 (https://www.aicrowd.com/challenges/global-wheat-challenge-2021). The validation set and the test set correspond to the partial leaderboard and the final leaderboard, respectively.

### 3.2. Overview of Dynamic Color Transform

**Figure 3:**
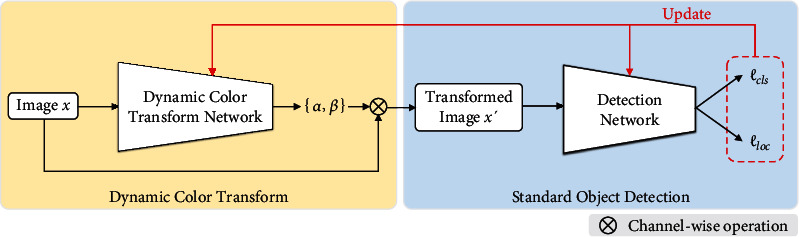
Dynamic color transform in a standard object detection pipeline. The input image **x** is first transformed to **x**′ by the DCT network, where **x**′ = *α ***x** + *β*. Then, **x**′ is sent to detection network for computing losses *ℓ*_*cls*_ and *ℓ*_*loc*_. The losses are used to update the DCT and the detection network.

Motivated by the observation that simple modification of color channels can improve detection results (Section 4.2), we propose a DCT network to improve wheat head detection. The use of the DCT network is depicted in Figure 3. Specifically, we first pass the input image **x** through the DCT network to obtain the transformed image **x**′. Then, we perform standard object detection to compute the loss and update the DCT and the detection network.

### 3.3. Color Transform Modeling

Due to different observation conditions, in-filed wheat head images would suffer from illuminations variations, which deteriorate the performance of the CNN models. In practice, illumination affects the contrast of color channels, suggesting that color is an important cue to tackle illumination variations. Therefore, we propose to model color transform by a DCT network. Sharing the same spirit of recent dynamic networks [22], DCT enables data-dependent inference. It dynamically generates the linear color transform parameters to modulate the color of the input image. An appealing property of DCT is that illumination variations can be corrected adaptively.

Given an input RGB image **x**, we adopt linear color transform to modulate **x** as follows:

(1) $$\begin{array}{c} \left(\begin{array}{c} R^{\prime }=\alpha_{R}R+\beta_{R},\\ G^{\prime }=\alpha_{G}G+\beta_{G},\\ B^{\prime }=\alpha_{B}B+\beta_{B}, \end{array}\right. \end{array}$$

where **R**, **G**, and **B** denote the red, green, and blue color channels of the input image **x**, respectively. **R**′, **G**′, and **B**′ are transformed color channels. *α*_*R*_, *α*_*G*_, *α*_*B*_, *β*_*R*_, *β*_*G*_, and *β*_*B*_ are color transform parameters predicted by the DCT network. Although these parameters can be modeled independently, we empirically find that it is better to unify the parameters of different color channels, i.e., *α*_*R*_, *α*_*G*_, and *α*_*B*_ share the same *α* and *β*_*R*_, *β*_*G*_, and *β*_*B*_ share the same *β*.

Formally, a DCT network *ϕ* parameterized by *θ* is applied to the input image **x**, predicting color transform parameters {*α*, *β*} by

(2) $$\begin{array}{c} \left\{\alpha ,\beta \right\}=\phi_{\theta }\left(x\right). \end{array}$$

Combining Equation (1), the transformed input image **x**′ can be written as

(3) $$\begin{array}{c} x^{\prime }=\alpha \cdot x+\beta , \end{array}$$

where **α** = [*α*, *α*, *α*], **β** = [*β*, *β*, *β*], and · denote channel-wise multiplication.

#### 3.3.1. Predicting *α* and *β*

Here, we present two formulations to predict *α* and *β*, including a regression-based formulation and a classification-based formulation.

*(1) Regression-Based Formulation*. Regression is the most intuitive way to predict *α* and *β*. Let *α*_**x**_ and *β*_**x**_ denote the outputs of the DCT network. We obtain *α* and *β* by

(4) $$\begin{array}{c} \alpha =\alpha_{\max}\cdot \text{Sigmoid}\left(\alpha_{x}\right), \end{array}$$

(5) $$\begin{array}{c} \beta =\beta_{\max}\cdot \frac{\arctan\left(\beta_{x}\right)}{\pi }, \end{array}$$

where *α*_max_ and *β*_max_ are hyperparameters that control the value range of *α* and *β*, respectively. Sigmoid is the sigmoid function, arctan is the inverse tangent function, and *π* is a mathematical constant defined as the ratio of a circle's circumference to its diameter. Note that *α* and *β* are in the range of (0, *α*_max_) and (−*β*_max_, *β*_max_), respectively.

*(2) Classification-Based Formulation*. We also present a classification-based idea to predict *α* and *β*. The motivation behind this is that we consider that classification may be easier to learn than regression. Specifically, we parameterize the values of color transform parameters by a discrete interval:

(6) $$\begin{array}{c} V_{\alpha }=\left\{i_{\alpha }\cdot k\;|\;k\in \left\{1,2,\cdots ,k_{\max}\right\}\right\}, \end{array}$$

(7) $$\begin{array}{c} V_{\beta }=\left\{i_{\beta }\cdot j\;|\;j\in \left\{-j_{\max},\cdots ,-1,0,1,\cdots ,j_{\max}\right\}\right\}, \end{array}$$

where *i*_*α*_ and *i*_*β*_ are step sizes, while *k*_max_ and *j*_max_ control the value range. For example, *V*_*α*_ = {0.1,0.2, ⋯, 1.0} if we set *i*_*α*_ = 0.1 and *k*_max_ = 10. With the definitions above, we use the DCT network to predict the probability of each element in *V*_*α*_ and *V*_*β*_, obtaining the color transform parameters by

(8) $$\begin{array}{c} \alpha =\overset{k_{\max}}{\underset{k=1}{\sum }}\;i_{\alpha }\cdot k\cdot p_{\alpha }\left(k\;|\;x\right), \end{array}$$

(9) $$\begin{array}{c} \beta =\overset{j_{\max}}{\underset{j=-j_{\max}}{\sum }}\;i_{\beta }\cdot j\cdot p_{\beta }\left(j\;|\;x\right), \end{array}$$

where *p*_*α*_(*k* | **x**) and *p*_*β*_(*j* | **x**) are the probability output of the DCT network. Note that ∑_*k*=1_^*k*_max_^ *p*_*α*_(*k* | **x**) = 1 and ∑_*j*=−*j*_max__^*j*_max_^ *p*_*β*_(*j* | **x**) = 1.

#### 3.3.2. DCT Network Architecture

Practically, DCT can be easily implemented as a dynamic network [21, 22]. Since off-the-shelf networks exhibit superior performance on computer vision tasks, in this work, we evaluate four different network architectures: ShuffleNetV2 [23], MobileNetV2 [43], ResNet18 [16], and ResNet34 [16]. The first two networks are lightweight and efficient, which have relatively low model capacity. On the contrary, ResNet18 is a medium-capacity model and ResNet34 is a high-capacity model. Note that the structure of the DCT network is not limited to existing networks, and one may manually design a DCT network.

**Figure 4:**
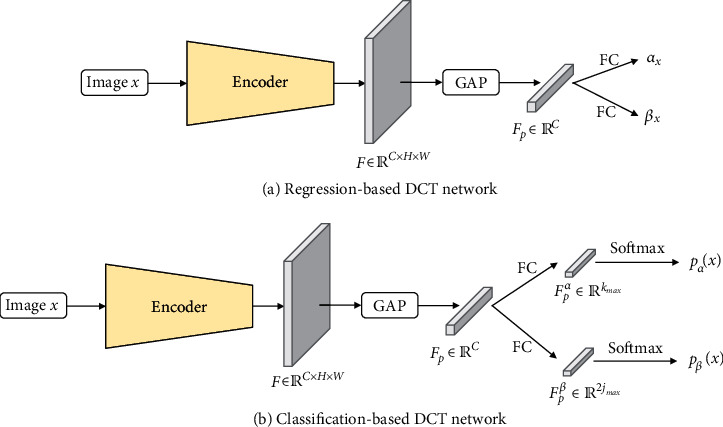
Illustration of regression-based DCT network and classification-based DCT network. GAP: Global Average Pooling; FC: fully connected layer; Softmax: softmax function.

Let the output features of the encoder of DCT network be denoted by *F* ∈ ℝ^*C*×*H*×*W*^, where *C*, *H*, and *W* are the channel number, height, and width of *F*, respectively. Following the modern CNN design protocol [16], we apply Global Average Pooling (GAP) on *F* to obtain the pooled feature *F*_*p*_ ∈ ℝ^*C*^. Next, we attach a fully connected layer to *F*_*p*_ to predict *α* and *β*.  Figure 4 illustrates the details of the regression-based DCT network and classification-based DCT network. For regression-based DCT, we directly output 2 parameters, i.e., *α*_**x**_ and *β*_**x**_. We then predict *α* and *β* following Equations (4) and (5). Regarding classification-based DCT, we first obtain intermediate representation *F*_*p*_^*α*^ ∈ ℝ^*k*_max_^ and *F*_*p*_^*β*^ ∈ ℝ^2*j*_max_^. Then, we apply the softmax function on *F*_*p*_^*α*^ and *F*_*p*_^*β*^, outputting the probability vector **p**_*α*_(**x**) and **p**_*β*_(**x**). *α* and *β* are subsequently computed via Equations (8) and (9).

### 3.4. Baseline Object Detector

We adopt a state-of-the-art object detector—Scaled-YOLOv4 [15]—as our baseline, which is the latest version of the YOLO series object detector [12, 27, 28, 30]. The reasons why we chose Scaled-YOLOv4 include the following:

1. It reports strong performance on generic object detection
2. It is clean to enable flexible modifications

More importantly, we empirically find that Scaled-YOLOv4 performs favorably against state-of-the-art methods on the GWHD 2021 dataset.  Table 1 shows the comparison results.

**Figure 5:**
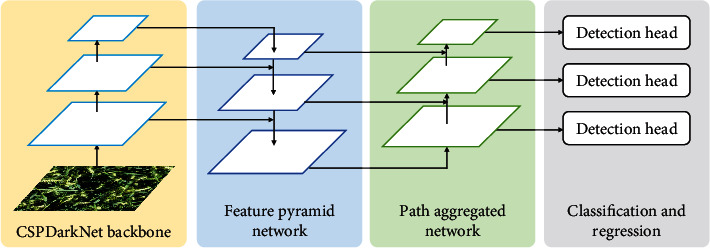
The architecture of Scaled-YOLOv4. It includes four parts: CSPDarkNet backbone, feature pyramid network, path aggregated network, and detection head.

Here, we briefly introduce the Scaled-YOLOv4 for the sake of completeness. The architecture of Scaled-YOLOv4 is illustrated in Figure 5. Multiscale features are first extracted by CSPDarkNet backbone. Feature pyramid network (FPN) and path aggregated network are then adopted to strengthen the representation capability of features. Finally, detection heads are deployed to predict objects.

*CSPDarkNet backbone*. Following YOLOv4, CSPDarkNet is adopted as the backbone network. CSP [18] tackles the heavy inference computations from the perspective of network architecture. It integrates features from the beginning and the end of a network stage, reducing computation cost by 20%. The advantages of CSPDarkNet are multifold: (i) it strengths the learning ability of a CNN; (ii) the amount of computation is evenly distributed at each layer in CNN, which removes computational bottlenecks by a significant magnitude; (iii) it reduces the memory cost, enabling efficient inference.

*Feature pyramid network (FPN)*. Feature pyramids are a classic idea in computer vision to address objects at different scales. To exploit the inherent pyramidal hierarchy of CNN, feature pyramid network is deployed. By building a top-down architecture with lateral connections, FPN can obtain high-level semantic features at multiple scales, which significantly improves the feature representation and benefits object detection.

*Path aggregated network (PAN)*. Information propagation is of great importance in CNN. The path aggregated network is applied to boost information flow. In contrast to FPN that is a top-down architecture, PAN adopts bottom-up path augmentation. In particular, it shortens the information path from low-level structure to topmost features. The accurate localization signals in low-level features are naturally propagated through the bottom-up path, enhancing the feature hierarchy.

*Detection head*. A detection head consists of classification and bounding box regression. The classification branch is attached to each PAN level, predicting the classes of each anchor box from multiple scales. Binary cross-entropy loss is adopted as a supervision signal. Parallel to the classification branch, the box regression branch predicts 4 coordinates for each box along with an objectness score. The objectness equals 1 if the anchor box overlaps with a ground-truth box more than any other anchor boxes. In addition, an anchor box that is not assigned with a ground-truth box contributes no loss for regression and classification. Note that a generalized intersection-over-union (GIoU) loss [45] is adopted as regression loss. GIoU loss breaks the gap between network training objective and metric evaluation by directly optimizing the metric itself, thus bringing consistent improvements in detection performance.

### 3.5. Loss Function

Given an object detector *f* parameterized by *ω* and the transformed input image **x**′, the training loss is formulated as

(10) $$\begin{array}{c} \underset{\theta ,\omega }{\min}L\left(f_{\omega }\left(x^{\prime }\right),\left\{y_{i},b_{i}\right\}\right), \end{array}$$

where {*y*_*i*_, **b**_*i*_} is the ground-truth label (*y*_*i*_ is the class label and **b**_*i*_ is the bounding box). In practice, *ℒ* is composed of classification loss and localization loss [12, 15]. Thus, Equation (10) can be rewritten as follows:

(11) $$\begin{array}{c} \underset{\theta ,\omega }{\min}\ell_{cls}\left(f_{\omega }\left(x^{\prime }\right),\left\{y_{i},b_{i}\right\}\right)+\ell_{loc}\left(f_{\omega }\left(x^{\prime }\right),\left\{y_{i},b_{i}\right\}\right), \end{array}$$

where *ℓ*_*cls*_ is a classification loss (i.e., cross-entropy loss) and *ℓ*_*loc*_ is a localization loss (i.e., GIoU loss [45]).

It is worth mentioning that our DCT network is not limited to specific object detectors. Here, we only instantiate an application of the DCT network on Scaled-YOLOv4 [15].

### 3.6. Implementation Details

#### 3.6.1. The Hyperparameters of the DCT Network

Since we present two formulations of DCT, i.e., regression-based DCT and classification-based DCT, here, we delineate their hyperparameters separately. For regression-based DCT, we set *α*_max_ = 2 and *β*_max_ = 0.1. *α* and *β* therefore are in the range of (0, 2) and (−0.1,0.1), respectively. For classification-based DCT, the hyperparameters are set as *i*_*α*_ = 0.1, *k*_max_ = 20, *i*_*β*_ = 0.1, and *j*_max_ = 2. The value range of *α* and *β* thus are [0.1,2] and [−0.2,0.2], respectively. Unless otherwise noted, we adopt ResNet18 as the DCT network.

#### 3.6.2. Training Details

We adopt a two-step training strategy, i.e., we first train the detection network, then we fix it and train the DCT network. Following [15], the detection network is trained for 300 epochs. The initial learning rate is set to 0.1, decaying with a cosine annealing schedule. Note that the input image is normalized to the range of [0, 1], which is the same as [15]. We employ heavy data augmentation to increase the diversity of training samples, including random scaling, random translation, random color distortion, random flip, and mosaic [12]. Regarding the DCT network, we train it for 50 epochs. We set the initial learning rate as 0.02, which is decreased by a factor of 10 every 20 epochs. Stochastic Gradient Descent (SGD) is adopted as the optimizer.

#### 3.6.3. Testing

To further improve the detection performance, we propose a voting-based model ensemble (VME) method.

**Figure 6:**
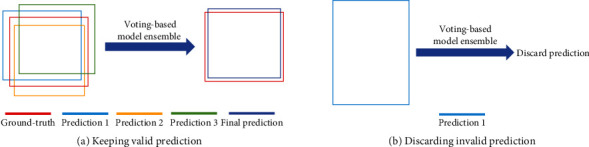
Illustration of two situations of the voting-based model ensemble, where *K* = 3. (a) There are three similar predictions, i.e., the number of predictions is more than [*K*/2]. Therefore, we average them to obtain the final prediction. (b) We discard the prediction because there only exists one prediction (the number of predictions is less than [*K*/2].

*(1) Voting-Based Model Ensemble*. For each image, suppose we are given a set of predictions {*ℬ*^*i*^}_*i*=1_^*K*^, where *ℬ*^*i*^ is the predictions of a model and *K* is the total number of different models. Our goal is to obtain better results by ensembling them. Let us denote one predicted box as *b*_*j*_^*i*^, where *i* ∈ {1, ⋯, *K*} and *j* ∈ {1, ⋯, *N*_*i*_} (*N*_*i*_ is the number of boxes in *ℬ*^*i*^). For each *b*_*j*_^*i*^, we keep it only when there are more than [*K*/2] similar boxes, i.e., *b*_*j*_^*i*^ is valid only when most models agree with it, otherwise discarded. Note that we consider that two boxes are similar when the intersection over union (IoU) between them is larger than a threshold *θ* (we set *θ* = 0.6). Among similar boxes, we further average them to reduce redundant boxes. In this way, we can obtain more accurate predictions and alleviate false positives.  Figure 6 illustrates two situations of VME. In particular, we use test time augmentation [46] (e.g., up-down flip, left-right flip, and rotation) to obtain the predictions set {*ℬ*^*i*^}_*i*=1_^*K*^.

In addition, we also use pseudolabeling [47] to achieve top ranking on GWC 2021 (Section 4.4), i.e., we retrain the model with a fusion of the training and testing data, where the predictions of our model on the test set are treated as pseudolabels.

## 4. Results

### 4.1. Evaluation Metric

We use Average Domain Accuracy (ADA) as the evaluation metric. The accuracy of each image is calculated by

(12) $$\begin{array}{c} \text{Accurac}{\text{y}}_{\text{image}}=\frac{\text{TP}}{\text{TP}+\text{FN}+\text{FP}}, \end{array}$$

where TP, FN, and FP are true positive, false negative, and false positive, respectively. A ground-truth box is considered to match with one predicted box if their IoU is higher than a threshold of 0.5. The accuracy of all images from the same domain is averaged to obtain the domain accuracy. The ADA is the average of all domain accuracy.

### 4.2. Impact of the Color Channel

**Figure 7:**
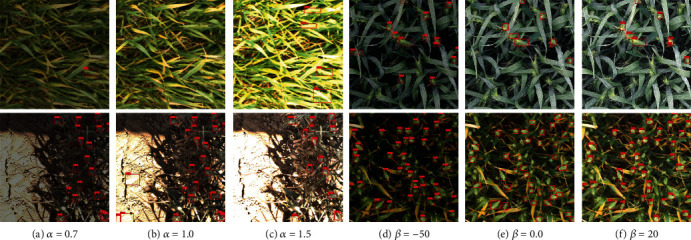
Qualitative results of Scaled-YOLOv4 [15] under different *α*'s and *β*'s. For (a–c), *β* is fixed to 0. For (d–f), we set *α* = 1.0. The numbers above the red detection boxes are the confidence scores. Best viewed by zooming in.

Here, we empirically investigate the impact of the color channel on wheat head detection and show that an appropriate treatment of color can improve detection. Specifically, given an object detector trained on the GWHD 2021 [7] dataset (e.g., we adopt Scaled-YOLOv4 [15]), we manually modify the value of each color channel using Equation (1), where *α*_*R*_ = *α*_*G*_ = *α*_*B*_ = *α* and *β*_*R*_ = *β*_*G*_ = *β*_*B*_ = *β*. We first fix *β* = 0 and vary *α* (*α* ∈ {0.7,1.0,1.5}). The qualitative results are shown in Figures 7(a)–7(c). Note that, the transformed image is the same as the original image when *α* = 1.0 and *β* = 0. Interestingly, we observe that modifying *α* can improve the detection results. For instance, false negatives are alleviated and false positives are suppressed. Next, we fix *α* = 1.0 and vary *β* (*β* ∈ {−50, 0, 20}). Figures 7(d)–7(f) show the qualitative results. Similarly, modifying the value of *β* can also improve detection.

**Figure 8:**
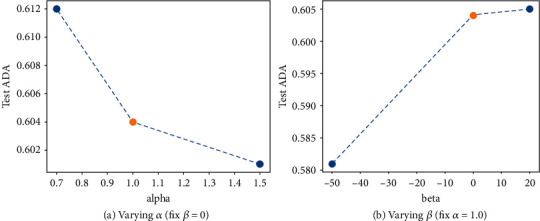
The test ADA plots of Scaled-YOLOv4 under different *α*'s and *β*'s on the GWHD 2021 test set. The orange point denotes baseline.

Moreover, we also compare the detection performance of Scaled-YOLOv4 under different *α*'s and *β*'s on the GWHD 2021 test set.  Figure 8 shows the test ADA plots of Scaled-YOLOv4, where the orange point (*α* = 1.0 and *β* = 0) denotes the baseline. We notice that an appropriate choice of *α* and *β* can indeed improve the ADA metric. For example, setting *α* = 0.7 improves the ADA from 0.604 to 0.612. The results in Figure 8 are consistent with the observation in Figure 7.

To summarize, our results indicate that color is a useful clue in wheat head detection. However, we remark that, despite color being useful, it is not sufficient to tackle object detection based on colors solely. The reasons are twofold:

1. Since wheat heads vary significantly in different domains, color information is not shared among different areas
2. Color is sensitive to observation/illumination conditions; thus, color distortions may occur when perturbation appears

Therefore, we relieve the role of the color and incorporate color information into existing object detectors to improve detection.

### 4.3. Ablation Study

#### 4.3.1. Effectiveness of DCT

**Figure 9:**
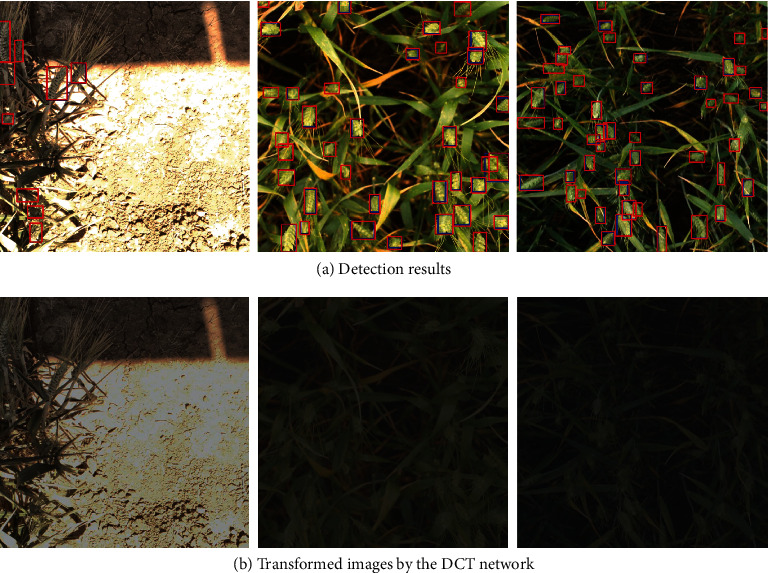
Visualization of detection results and transformed images. The predictions of our DCT Scaled-YOLOv4 are in red. The results of the baseline Scaled-YOLOv4 (without DCT) are in blue.

Table 2 shows the comparison results of baseline Scaled-YOLOv4 and DCT Scaled-YOLOv4, where Val ADA and Test ADA denote the ADA on validation and test sets, respectively. Regression-based DCT and classification-based DCT both achieve notable improvements over baseline, which validates the effectiveness of our approach. Specifically, the former boosts the baseline from 0.604 to 0.629 on the test set. The latter obtains similar results on the test set, with an ADA of 0.630. Note that the validation set has low illumination variations; therefore, our DCT only achieves minor improvements on the validation set. Since the results between our DCT and baseline are relatively close in Val ADA, we repeat the experiments three times with different random seeds, aiming to confirm that our higher results are not due to chance.  Table 3 shows the detailed results. For classification-based DCT, the results of Val ADA are 0.78233 ± 0.00047, where 0.78233 is the mean ADA and 0.00047 is the standard deviation. Regarding regression-based DCT, it achieves a mean Val ADA of 0.78500 and a standard deviation of 0.00141. The results above imply that our DCT indeed brings consistent improvements over baseline instead of by chance. To further understand the impact of DCT, we visualize the detection results and the transformed image in Figure 9. The DCT model is robust to various illumination conditions and performs consistently better than standard Scaled-YOLOv4. For example, it significantly reduces the number of false negatives.

#### 4.3.2. Comparison of Different DCT Networks

Table 4 compares the performance of different DCT backbones, including ShuffleNetV2 [23], MobileNetV2 [43], ResNet18 [16], and ResNet34 [16]. Our results indicate that regression-based DCT and classification-based DCT are both robust to the choice of backbone networks. Among them, ResNet18 achieves the best performance. Notice that applying lightweight networks are sufficient to achieve good performance. For example, ShuffleNetV2 only has 0.8 M and 1.0 M parameters in regression-based DCT and classification-based DCT, respectively. With negligible overhead parameters, it achieves competing results against ResNet18 DCT. In addition, it is worth mentioning that the inference time of ResNet18 DCT network is 7 ms on a single RTX 3090 GPU (i.e., around 142 frames per second), indicating that the DCT network is efficient.

#### 4.3.3. Sensitiveness of DCT Parameters

Here, we investigate the sensitiveness of the hyperparameters in regression-based DCT and classification-based DCT.

*Sensitiveness of regression-based DCT*. We manually tune *α*_max_ and *β*_max_ to examine the sensitiveness of regression-based DCT.  Table 5 shows the detailed results. Increasing the range of *α* from (0, 2) to (0, 3) slightly degrades the detection performance, which suggests that *α* is not necessary to have a large value range. Similarly, extending the range of *β* from (−0.1,0.1) to (−0.2,0.2) does not bring further improvement. Nevertheless, the above results demonstrate that regression-based DCT is not sensitive to hyperparameters. In addition, we recommend to use relatively small *α*_max_ and *β*_max_, e.g., *α*_max_ = 2 and *β*_max_ = 0.1 already achieve good performance.

*Sensitiveness of classification-based DCT*. Since the hyperparameters of classification-based DCT control discrete interval *V*_*α*_ and *V*_*β*_, we separately investigate their effects. To show the sensitiveness of *V*_*α*_, we adopt three different configurations, resulting in various *V*_*α*_ and *α* ranges. Note that we limit the maximum value of *α* to 2 and set *β* = 0.  Table 6 indicates that classification-based DCT is relatively robust to different *V*_*α*_. The best results are achieved when interval *i*_*α*_ = 0.1, suggesting that we shall choose an appropriate interval value. Coarse interval (*i*_*α*_ = 0.2) may miss the optimal *α* value, while fine interval (*i*_*α*_ = 0.05) may confuse the classification model. Therefore, both of them lead to suboptimal results.

For *V*_*β*_, we experiment with four different configurations, where we fix *V*_*α*_ = {0.1,0.2, ⋯, 2.0}. The results in Table 6 show that classification-based DCT is also robust to the choice of *V*_*β*_. We observe that it is not necessary to use a too-small interval (e.g., *i*_*β*_ = 0.02). In addition, the range of *β* has a minor impact on detection performance.

#### 4.3.4. Effectiveness of VME

**Figure 10:**
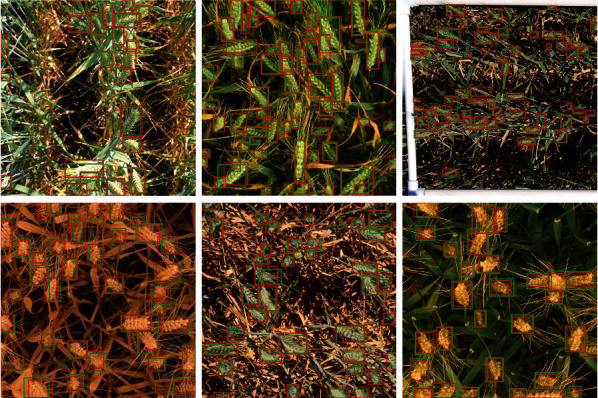
Qualitative results of our method on the GWHD 2021 validation and test sets. The predictions are in red, and the ground-truth labels are in green.

The comparison results are shown in Table 7. Applying VME further unveils the potential of our approach. For the regression-based DCT model, it improves ADA by 0.9% and 2.8% on validation and test sets, respectively. The best performance is achieved by the classification-based DCT model with VME, with Val ADA of 0.802 and Test ADA of 0.657.  Figure 10 shows the qualitative results on the GWHD 2021 dataset. The predictions are in red, and the ground-truth labels are in green. It is worth noticing that our method achieves pleasing results under various illumination conditions.

### 4.4. Qualitative Results on the Global Wheat Challenge 2021

We participated in the GWC 2021 using our method. The competition results are shown in Table 8, and the username of our team is SMART. We rank second in the final leaderboard, with an ADA of 0.695. In addition, we rank first in the partial leaderboard (i.e., initial public leaderboard), with an ADA of 0.821. Here, we only show the results of the top 10 teams. We refer readers to the leaderboard page (https://www.aicrowd.com/challenges/global-wheat-challenge-2021/leaderboards) for full results. Note that, despite GWC 2021 and GWHD 2021 sharing the same data, the results of our method in Table 1 are different from those in Table 8. The reasons are twofold: (1) we ensemble the predictions of multiple models in GWC 2021 to obtain top ranking, but we report the results of a single model in GWHD 2021 for fair comparison; (2) we also adopt pseudolabeling [47] to improve the detection performance in GWC 2021.

## 5. Discussion and Conclusion

In this work, we introduce a simple but effective idea—dynamic color transform—for wheat head detection. By incorporating our DCT network into an existing object detector, we observe a notable improvement in wheat head detection. The DCT network exhibits robustness to various illumination conditions and indicates that a simple idea can make a difference if it is treated the right way. Our method reports state-of-the-art results on the GWHD 2021 dataset and achieves runner-up performance on the GWC 2021.

In the experimental section, we empirically investigate the design of DCT networks, the choice of DCT networks, and the sensitiveness of hyperparameters (the range of *α* and *β*). Our results show the following: (i) Regression-based DCT and classification-based DCT are both applicable to wheat head detection. In addition, the latter performs slightly better when applying VME during testing. (ii) The performance of DCT is robust to the backbone networks chosen, and lightweight networks are sufficient to work. (iii) The DCT is not sensitive to hyperparameters. (iv) The DCT is efficient and reports state-of-the-art results with negligible overhead parameters.

Although DCT has performed favorably on the GWHD 2021 dataset, there still exist several limitations. First, it is difficult for our model to distinguish objects that have similar colors to the backgrounds. We infer that the global color transform deployed by our DCT could not tackle well similar objects and background. Local DCT may be an alternative choice to address this problem. In addition, blurred images may also render detection failure. Second, DCT is helpful when dealing with illumination variations. The impact of DCT may be minor when images are captured under a constant illumination condition.

For future work, we intend to extend our method to other plant detection tasks, e.g., maize tassel detection.

## Figures and Tables

**Table 1:** Comparison results of several state-of-the-art methods on the GWHD 2021 validation and test sets. The evaluation metric is ADA (see Section 4.1 for the definition of ADA).

Method	Val ADA	Test ADA
FasterRCNN [13]	0.632	0.511
FCOS [44]	0.731	0.554
Scaled-YOLOv4 [15]	0.777	0.604

**Table 2:** Ablation study of our DCT on the GWHD 2021 validation and test sets. The evaluation metric is ADA.

Method	Val ADA	Test ADA
Baseline	0.777	0.604
Baseline+regression-based DCT	0.787	0.629
Baseline+classification-based DCT	0.782	0.630

**Table 3:** Random seeds experiments of our DCT on the GWHD 2021 validation and test sets. The results (mean ± std) are reported over 3 runs with different random seeds.

Method	Val ADA	Test ADA
Regression-based DCT	0.78500 ± 0.00141	0.62733 ± 0.00125
Classification-based DCT	0.78233 ± 0.00047	0.62800 ± 0.00163

**Table 4:** Comparison of different DCT networks on the GWHD 2021 validation and test sets; the evaluation metric is ADA.

DCT network	Regression-based DCT			Classification-based DCT		
	Parameters	Val ADA	Test ADA	Parameters	Val ADA	Test ADA
ShuffleNetV2 [23]	0.8 M	0.781	0.626	1.0 M	0.782	0.628
MobileNetV2 [43]	2.2 M	0.782	0.629	3.9 M	0.780	0.630
ResNet18 [16]	11.2 M	0.787	0.629	11.4 M	0.782	0.630
ResNet34 [16]	21.3 M	0.781	0.622	21.6 M	0.781	0.629

**Table 5:** Sensitiveness of regression-based DCT.

Hyperparameters		*α* range	*β* range	Val ADA	Test ADA
*α*_max_	*β*_max_				
2	0.2	(0, 2)	(-0.2, 0.2)	0.785	0.626
2	0.1	(0, 2)	(-0.1, 0.1)	0.787	0.629
3	0.1	(0, 3)	(-0.1, 0.1)	0.784	0.623

**Table 6:** Sensitiveness of classification-based DCT.

Variable	Hyperparameters		*V*_*α*_ (Equation (6))	*α* range	Val ADA	Test ADA
	*i*_*α*_	*k*_max_				
*α*	0.05	40	{0.05,0.1, ⋯, 2.0}	[0.05,2]	0.779	0.626
	0.1	20	{0.1,0.2, ⋯, 2.0}	[0.1,2]	0.780	0.628
	0.2	10	{0.2,0.4, ⋯, 2.0}	[0.2,2]	0.777	0.623

Variable	Hyperparameters		*V*_*β*_ (Equation (7))	*β* range	Val ADA	Test ADA
	*i*_*β*_	*j*_max_				
*β*	0.02	5	{−0.1, −0.08, ⋯, 0.1}	[-0.1,0.1]	0.781	0.628
	0.05	2	{−0.1, −0.05, ⋯, 0.1}	[-0.1,0.1]	0.781	0.629
	0.05	4	{−0.2, −0.15, ⋯, 0.2}	[-0.2,0.2]	0.778	0.629
	0.1	2	{−0.2,0.1, ⋯, 0.2}	[-0.2,0.2]	0.782	0.630

**Table 7:** Ablation study of VME on the GWHD 2021 validation and test sets. The evaluation metric is ADA.

Method	Val ADA	Test ADA
Reg. DCT	0.787	0.629
Reg. DCT+VME	0.796	0.657
Cls. DCT	0.782	0.630
Cls. DCT+VME	0.802	0.657

Reg. DCT: regression-based DCT; Cls. DCT: classification-based DCT.

**Table 8:** Final and partial leaderboard of the Global Wheat Challenge 2021.

Final leaderboard			Partial leaderboard		
Rank	Participants	ADA	Rank	Participants	ADA
1	randomTeamName	0.700	1	SMART	0.821
2	SMART	0.695	2	Kosung	0.812
3	david_jeon	0.695	3	wheat_hunters	0.811
4	keyhan_najafian	0.692	4	randomTeamName	0.807
5	hitsz	0.689	5	david_jeon	0.807
6	maxim	0.682	6	Hitsz	0.805
7	kosung	0.676	7	augly_wheat	0.792
8	augly_wheat	0.671	8	Wu_Chun_Huan_	0.790
9	Wu_Chun_Huan_	0.669	9	UoL	0.787
10	Ural	0.666	10	vlad_barbu	0.786

## Data Availability

The GWHD 2021 dataset is available at https://zenodo.org/record/5092309.
